# Interferometric diffuse correlation spectroscopy improves measurements at long source–detector separation and low photon count rate

**DOI:** 10.1117/1.JBO.25.9.097004

**Published:** 2020-09-30

**Authors:** Mitchell B. Robinson, David A. Boas, Sava Sakadzic, Maria Angela Franceschini, Stefan A. Carp

**Affiliations:** a Harvard Medical School, Massachusetts General Hospital, Optics at Athinoula A. Martinos Center for Biomedical Imaging, Charlestown, Massachusetts, United States; b Massachusetts Institute of Technology, Harvard-MIT Program in Health Sciences and Technology, Cambridge, Massachusetts, United States; c Boston University, Boston University Neurophotonics Center, Boston, Massachusetts, United States

**Keywords:** diffuse correlation spectroscopy, speckle interferometry, biomedical optics, spectroscopy, medical imaging

## Abstract

**Significance:** The use of diffuse correlation spectroscopy (DCS) has shown efficacy in research studies as a technique capable of noninvasively monitoring blood flow in tissue with applications in neuromonitoring, exercise science, and breast cancer management. The ability of DCS to resolve blood flow in these tissues is related to the optical sensitivity and signal-to-noise ratio (SNR) of the measurements, which in some cases, particularly adult cerebral blood flow measurements, is inadequate in a significant portion of the population. Improvements to DCS sensitivity and SNR could allow for greater clinical translation of this technique.

**Aim:** Interferometric diffuse correlation spectroscopy (iDCS) was characterized and compared to traditional homodyne DCS to determine possible benefits of utilizing heterodyne detection.

**Approach:** An iDCS system was constructed by modifying a homodyne DCS system with fused fiber couplers to create a Mach–Zehnder interferometer. Comparisons between homodyne and heterodyne detection were performed using an intralipid phantom characterized at two extended source–detector separations (2.4, 3.6 cm), different photon count rates, and a range of reference arm power levels. Characterization of the iDCS signal mixing was compared to theory. Precision of the estimation of the diffusion coefficient and SNR of the autocorrelation curve were compared between different measurement conditions that mimicked what would be seen *in vivo*.

**Results:** The mixture of signals present in the heterodyne autocorrelation function was found to agree with the derived theory and resulted in accurate measurement of the diffusion coefficient of the phantom. Improvement of the SNR of the autocorrelation curve up to ∼2× and up to 80% reduction in the variability of the diffusion coefficient fit were observed for all measurement cases as a function of increased reference arm power.

**Conclusions:** iDCS has the potential to improve characterization of blood flow in tissue at extended source–detector separations, enhancing depth sensitivity and SNR.

## 1 Introduction

Diffuse correlation spectroscopy (DCS) is an optical technique that measures the temporal fluctuations of multiply scattered light to characterize the displacement dynamics of scattering particles in the sample under interrogation.^1^ By analyzing the time course of the measured intensity signal through the intensity autocorrelation, $g_{2}(\tau )$, measurements of blood flow can be derived by utilizing the correlation diffusion theory.^2^ This technique has many benefits for clinical monitoring, including its noninvasive nature and its ability to allow for continuous monitoring. Blood flow measurements using DCS have been explored as a method to monitor cerebral blood flow at the bedside,^3^^–^^5^ to distinguishing benign and malignant breast cancer lesions,^6^^,^^7^ and to monitor skeletal muscle physiology in exercise science.^8^^–^^10^ In all areas of application, the presence of intervening superficial layers can distort the DCS blood flow measurements of the tissue of interest (extracerebral layers for head measurements, subcutaneous fat for muscle measurements, and noninvolved breast tissue for the breast measurements). In the neuromonitoring arena, one of the most promising application areas, clinical translation of DCS has yet to be realized despite the benefits of the technique having been shown in several clinical conditions, including stroke, traumatic brain injury, hydrocephalus, and Alzheimer’s disease.^11^^–^^14^ This in part is due to difficulties in cerebral blood flow measurements in adults due to the significant thickness of the extracerebral layers.^15^

The sensitivity to deep-seated blood flow can be increased by extending the source–detector separation used in the measurement. However, the use of a longer source–detector separation comes at the cost of signal-to-noise ratio (SNR), requiring longer averaging times to achieve the same blood flow precision and impeding some applications, including estimation of intracranial pressure via detection of pulsatile blood flow and functional activation.^13^^,^^16^^,^^17^ To overcome this challenge, we proposed the use of interferometric diffuse correlation spectroscopy (iDCS) to improve SNR and allow for longer source–detector separation measurements to improve sensitivity to blood flow at extended depths in tissue. This technique has been utilized to estimate cerebral blood flow in a shot-noise limited regime using a conventional CMOS camera.^18^ The work by Zhou et al. demonstrated the benefits of multiple pixel detection on SNR and showed that the technique is effective in measuring the blood flow index *in vivo*. Though the approach benefits from many aspects of the implementation, including parallel speckle detection, improving SNR by averaging, and insensitivity to room light, the hardware limitations imposed by the frame rate of available CMOS cameras limit the range of blood flow indices that can be measured and the use of multimode fiber, though enabling parallel speckle detection, introduces potential for the signals measured to be extremely sensitive to motion of the multimode fiber. The long source–detector separation is used to improve both the sensitivity to the blood flow in the tissue of interest versus blood flow in the superficial layers, and sensitivity to the higher blood flow present in the brain cortex, skeletal muscle, or malignant lesions. However, the longer photon path lengths accumulate phase changes faster due to scatterer motion and this causes a faster decay of the autocorrelation function. At 3 cm on a typical adult forehead, the measured electric field autocorrelation function $[g_{1}(\tau )\;]\;$ decays by 20% or more before the first time lag achievable by the camera (1/sampling rate=3  μs).^18^^,^^19^ Accurately sampling the decay of the autocorrelation at early lag times is valuable because it is more reflective of the photons that have traveled farther in the tissue (e.g., deeper in the tissue), offering increased sensitivity at depth.^15^ Here, we utilize the detectors traditionally used for homodyne DCS at traditional near-infrared spectroscopy (NIRS) wavelengths, silicon single-photon avalanche diodes (Si-SPADs), to allow for higher iDCS bandwidth measurements at an intermediate regime of reference arm intensity and we demonstrate the improved SNR of the heterodyne DCS measurements at large source–detector separation and low signal.

## 2 Methods and Materials

### 2.1 Homodyne DCS Theory

DCS measures the temporal speckle fluctuations collected from tissue samples. The autocorrelation of the intensity time course, $g_{2}(\tau )$, is related to the motion in the sample by the Siegert relation,^20^ given below in Eq. (1)

$$g_{2}(\tau )=1+\beta {\left|g_{1}(t)\right|}^{2},$$ (1)

where β is a coherence factor that is dependent upon the geometry of the measurement, reflectance versus transmission, laser coherence, and number of modes collected. The Siegert relation allows for the connection of observed intensity fluctuations to the underlying, normalized electric field autocorrelation function, $g_{1}(\tau )$. Fitting of the observed dynamics is then performed using the theoretically derived expression for $g_{1}(\tau )$, given by Eq. (2)^2^

$$G_{1}(\tau )=\int_{0}^{\infty }P(s)e^{-\frac{1}{3}k_{0}^{2}\langle \Delta r^{2}(\tau )\rangle \frac{s}{l^{*}}}ds.$$ (2)

In Eq. (2), $k_{0}$ is the wavenumber of the light, $l^{*}$ is the transport mean-free-path, $\left\langle \Delta r^{2}(\tau )\right\rangle$ is the mean-squared displacement of the light scattering particles at a delay τ, and s is the path length of the detected photons. The mean-squared displacement is given by $\langle \Delta r^{2}(\tau )\rangle =\alpha \cdot 6D_{b}\tau$, where we fit for the relevant parameter blood flow index (${\mathrm{BF}}_{i}$) as $\mathrm{BF}i=\alpha D_{b}$, where α is the fraction of scattering events that occur from moving scattering particles. Though one might expect a flow parameter representing multiply scattered, random flow, which decays on the order of $\tau^{2}$, it has been found that the ${\mathrm{BF}}_{i}$ measured *in vivo* is better fit by the diffusion coefficient.^21^^–^^23^

### 2.2 Heterodyne DCS Theory

Heterodyne (or interferometric) DCS utilizes a reference arm to coherently amplify the speckle fluctuations induced by the scatterer motion in the tissue.^24^ A heterodyne measurement involves a summation of the multiply scattered sample field, $E_{S}(t)$, and the reference arm electric field, $E_{R}(t)$, given as $E(t)=E_{S}(t)+E_{R}(t)$. The intensity of the detected light is then given by I(t)=[ES(t)ES*(t)]+[ER(t)ER*(t)]+2Re[ES(t)ER*(t)]. When the normalized intensity autocorrelation function, $g_{2}(\tau )$, is computed, the expanded form can be written as

$$g_{2}(\tau )=1+\beta_{0}[\frac{{\left\langle I_{S}(t)\right\rangle }^{2}}{{\left\langle I_{T}(t)\right\rangle }^{2}}g_{1}^{2}(\tau )+\frac{2\langle I_{S}(t)\rangle \langle I_{R}(t)\rangle }{{\left\langle I_{T}(t)\right\rangle }^{2}}g_{1}(\tau )],$$ (3)

where $\beta_{0}$ is the coherence parameter of the homodyne measurement, $\left\langle I_{S}(t)\right\rangle$ is the average of the sample arm intensity, $\left\langle I_{R}(t)\right\rangle$ is the average of the reference intensity, and $\left\langle I_{T}(t)\right\rangle$ is the sum of $\left\langle I_{S}(t)\right\rangle$ and $\left\langle I_{R}(t)\right\rangle$, representing the total average intensity. The two components of the heterodyne $g_{2}(\tau )$ can be seen to scale as a function of the reference intensity, and by rearrangement of the terms, as a function of the single variable, the fractional reference intensity, $I_{R}/I_{T}$, can be expressed as $\beta_{1}=\beta_{0}(1-\frac{I_{R}}{I_{T}}{)}^{2}$ and $\beta_{2}=2\beta_{0}\frac{I_{R}}{I_{T}}(1-\frac{I_{R}}{I_{T}})$, where $\beta_{1}$ and $\beta_{2}$ are the weighting coefficients for the quadratic and linear terms of $g_{1}(\tau )$, respectively, detailed below in Eq. (4). The rearrangement allows for independence of data analysis on the sample-dependent count rate, easing comparisons across different experimental conditions.

$$g_{2}(\tau )=1+\beta_{1}g_{1}^{2}(\tau )+\beta_{2}g_{1}(\tau )=1+\beta_{0}{\left(1-\frac{I_{R}}{I_{T}}\right)}^{2}g_{1}^{2}(\tau )+2\beta_{0}\frac{I_{R}}{I_{T}}(1-\frac{I_{R}}{I_{T}})g_{1}(\tau ).$$ (4)

### 2.3 Signal-to-Noise Ratio Considerations

The noise model for the homodyne intensity autocorrelation function provides guidance on the possible improvements achieved by utilizing a heterodyne approach.^25^ In Eq. (5), the noise of the autocorrelation curve is dependent upon the expected number of detected photons per time bin, ⟨n⟩, the averaging time, t, the bin time width, T, the single exponential, effective decay coefficient of the electric field autocorrelation function, Γ, the correlation lag time, τ, and the coherence parameter, β. Though the decay of the autocorrelation is more complex than a single exponential decay, this form of the decay $\left[g_{2}(\tau )\approx 1+\beta \;\exp{\left(\Gamma \tau \right)}^{2}\right]$ has been used previously to characterize the noise of the autocorrelation curve and was noted to not give statistically significant differences between estimated and measured noise of the curve.^26^

$$\sigma (\tau )=\sqrt{\frac{T}{t}}{\left[\beta^{2}\frac{(1+e^{-2\Gamma T})(1+e^{-2\Gamma \tau })+2\frac{\tau }{T}(1-e^{-2\Gamma T})e^{-2\Gamma \tau }}{1-e^{-2\Gamma T}}+2{\left\langle n\right\rangle }^{-1}\beta (1+e^{-2\Gamma \tau })+{\left\langle n\right\rangle }^{-2}(1+\beta e^{-\Gamma \tau })\right]}^{\frac{1}{2}}$$ (5)

Equation (5) was derived for the case of a homodyne DCS measurement, but it can be adapted to a reference-arm-dominated heterodyne measurement by substituting $\beta \to \beta_{0}\frac{2\langle n_{s}\rangle }{\left\langle n_{T}\right\rangle }$ and $\left\langle n\rangle \to \langle n_{T}\right\rangle$.^25^

The expression for the $g_{2}(\tau )$ noise [Eq. (5)] is complex, and ways to improve SNR are dependent upon factors that vary as a function of the sample measured, such as the photon count rate and the decay coefficient. To capture a wide range of correlation lags of the autocorrelation function, a logarithmically binned time array is typically used. This results in reduced noise at longer correlation lag times, τ, as time bins increase in duration and the average number of photons per bin, ⟨n⟩, increases. For most DCS measurements at early lag times ($\tau =10^{-7}\;to\;10^{-5}\;s$), ⟨n⟩ for a homodyne measurement is typically much <1, and the third term in Eq. (5) [e.g., ${\left\langle n\right\rangle }^{-2}(1+\beta e^{-\Gamma \tau })$] dominates the expression. The first term will dominate when ⟨n⟩≫1, and the second term typically falls somewhere between the first and the third. To evaluate how the noise of the autocorrelation curve in different measurement conditions affects the variability in the time trace of ${\mathrm{BF}}_{i}$, the coefficient of variation is calculated, given as $\sigma_{{\mathrm{BF}}_{i}}\;/\langle {\mathrm{BF}}_{i}\rangle$, where $\sigma_{{\mathrm{BF}}_{i}}$ is the standard deviation of the ${\mathrm{BF}}_{i}$ time course and $\left\langle {\mathrm{BF}}_{i}\right\rangle$ is the average fitted value of ${\mathrm{BF}}_{i}$.

### 2.4 Instrumentation

**Fig. 1:**
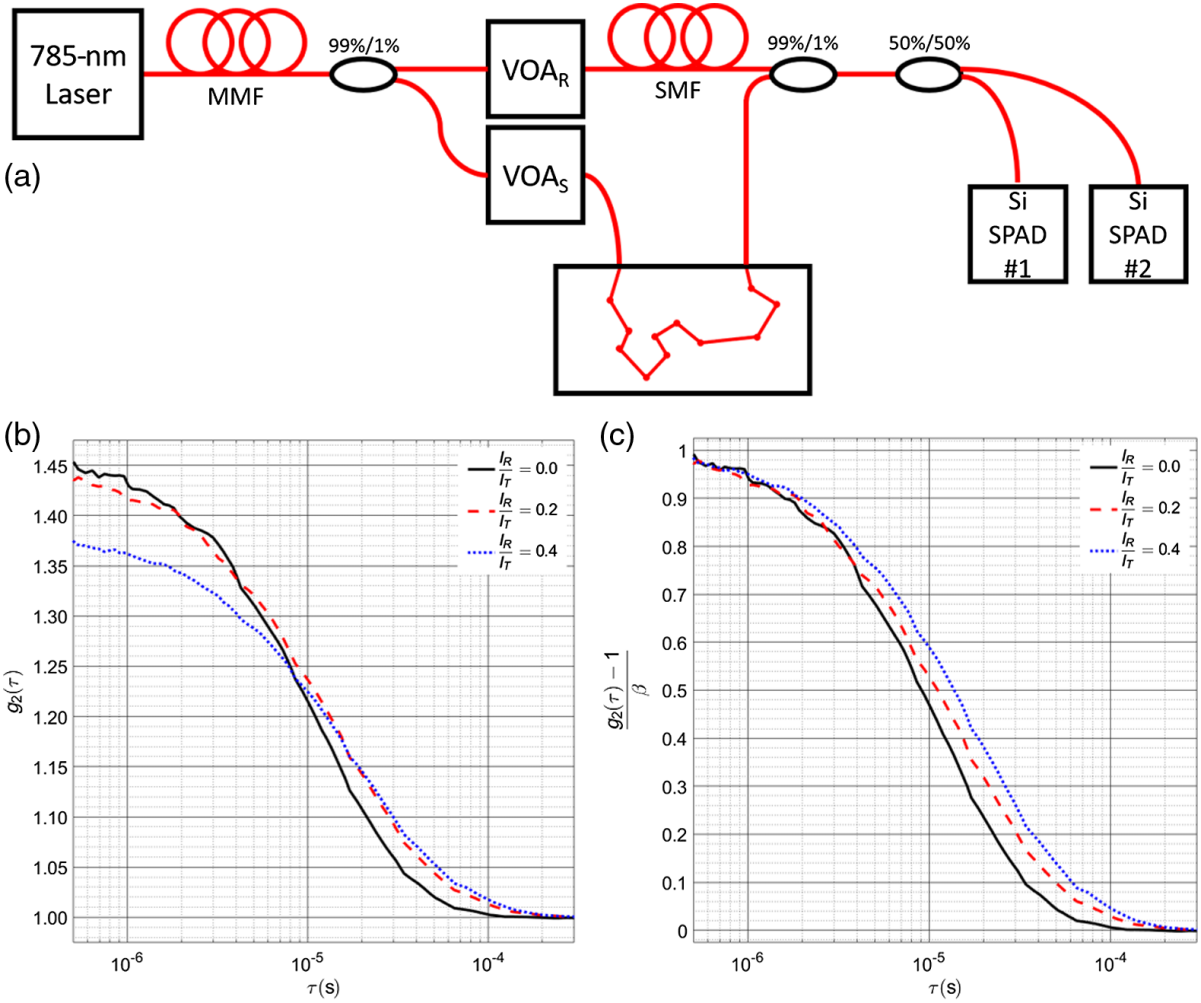
(a) The arrangement of the iDCS set up. The 785-nm light from a long coherence length laser (DL785-100-S, Crystalaser) is split between the sample arm (99%) and the reference arm (1%). A multimode variable attenuator (VOAMMF, Thorlabs) is used in the sample arm to vary the homodyne count rate for each measurement. A variable attenuator is placed in the reference arm (BB-100-11-780-5/125-S-50-3A3A-1-1, OZ Optics) to vary the fractional reference power. The multiply scattered light is recombined with the reference arm at a ratio of 99%/1%, and the combined signal is then split by a 50%/50% splitter to send to two SPAD detectors. (b) A sample of the intensity autocorrelation curves generated at different fractional reference intensities, showing differences in the achieved coherence parameter value, though when these differences in coherence parameter are normalized, (c) it can be seen that the decay rate changes as the weighting between the linear and quadratic terms of $g_{1}(\tau )$ are modulated as a function of the fractional reference intensity.

We used a custom-built DCS system consisting of a 785-nm laser (DL785-100-S, Crystalaser) and a multimode fused fiber coupler (99:1 coupling ratio, TM105R1F1A, Thorlabs) to split the light going to the sample (99%) and reference arms (1%). In both the sample and the reference arms, variable optical attenuators were used to independently vary the power going into these two arms. The dynamic phantom used in the experiment was made from a mixture of intralipid and water to reach optical properties, verified by an ISS MetaOx frequency domain NIRS instrument, of $\mu_{a}=0.03\;{\mathrm{cm}}^{-1}$, ${\mu_{s}}^{\prime }=6\;{\mathrm{cm}}^{-1}$, and $D_{b}=1.6\times 10^{-8}\;{\mathrm{cm}}^{2}/s$ at the room temperature of 20°C. The $D_{b}$ and ${\mu_{s}}^{\prime }$ of the phantom are similar to values reported in humans. The absorption of the phantom ($\mu_{a}=0.03\;{\mathrm{cm}}^{-1}$) was intentionally chosen to be lower than what might be observed in tissue measurements ($\mu_{a}=0.1$ to $0.2\;{\mathrm{cm}}^{-1}$),^27^ to allow the ability to explore different count rate regimes at large separations by modulating the source intensity, as though the light was attenuated by the tissue. The multiply scattered light was collected with a single-mode fiber and recombined with the reference arm using a fused fiber coupler (99:1 coupling ratio, TN785R1F2, Thorlabs). The signal was then divided into two equal channels using a single-mode fused fiber coupler (50:50 coupling ratio, TN785R5F2, Thorlabs) and detected by two Si-SPADs (SPCM-AQRH14, Excelitas). The use of dual detectors was intended to allow taking both autocorrelation and cross correlation measurements as shown later. Photon arrival times were recorded with a temporal resolution of 6.67 ns, and analysis of the timestamps was done during the postprocessing. A diagram of the system can be seen in Fig. 1(a).

### 2.5 Phantom Experiments

To explore the cases where iDCS improves SNR with respect to conventional homodyne DCS, we evaluated two decay regimes [e.g., two different decay rates of $g_{1}(\tau )$ simulating changes in the blood flow], and for each decay regime, we explored a range of intensities incident on the sample (and hence, count rates at the detector). Measurements were performed at the source–detector separations of 2.4 and 3.6 cm, which in our phantom resulted in decay times of $10^{-4}\;s$ and $10^{-5}\;s$ for the homodyne autocorrelation function to reach the level of ∼5% of the β parameter, respectively. The count rate in the homodyne measurement was adjusted to simulate different conditions of light attenuation and coupling. For the measurements taken at a 2.4-cm source–detector separation, the homodyne count rates used were 7.5, 15, and 30 kcps. For the measurements taken at a 3.6-cm source–detector separation, the homodyne count rates used were 5 and 10 kcps. The count rates explored at the given separations allowed comparisons of the iDCS improvement of SNR with signal levels that are likely to be seen in human measurements, where count rates are particularly low (5 and 10 kcps), and evaluate the relative weighting of the linear and quadratic terms of the heterodyne autocorrelation function with better measurement SNR (15 and 30 kcps) as a function of the reference intensity used. Table 1 summarizes the experimental conditions used in these experiments and how they will be referred to in the remainder of the text.

**Table 1:** Experimental conditions.

	Source–detector separation (cm)	Homodyne photon count rate (kcps)
Case 1	2.4	7.5
Case 2	2.4	15
Case 3	2.4	30
Case 4	3.6	5
Case 5	3.6	10

## 3 Results and Discussion

### 3.1 Characterizing the Weighting of the Terms in the Measured Intensity Autocorrelation

To evaluate the predictions made regarding the influence of the two components present in the measured autocorrelation function in the intermediate regime of iDCS, comparisons of the autocorrelation function and their fits were performed over a range of fractional reference intensities. A representative set of autocorrelation functions averaged from the 180-s measured intervals from case 3 (shorter separation 2.4 cm and highest count rate 30 kcps) is shown in Figs. 1(b) and 1(c), revealing the change in the value of the beta parameter as a function of fractional reference intensity (b) and the change in the decay rate (c). The qualitative comparison shown here follows well with the proposed model, as reference intensity increases, the sum of the beta parameters, $\beta_{1}$ and $\beta_{2}$, decreases, and the weighting of the linear term of $g_{1}(\tau )$ increases, slowing the overall decay.

**Fig. 2:**
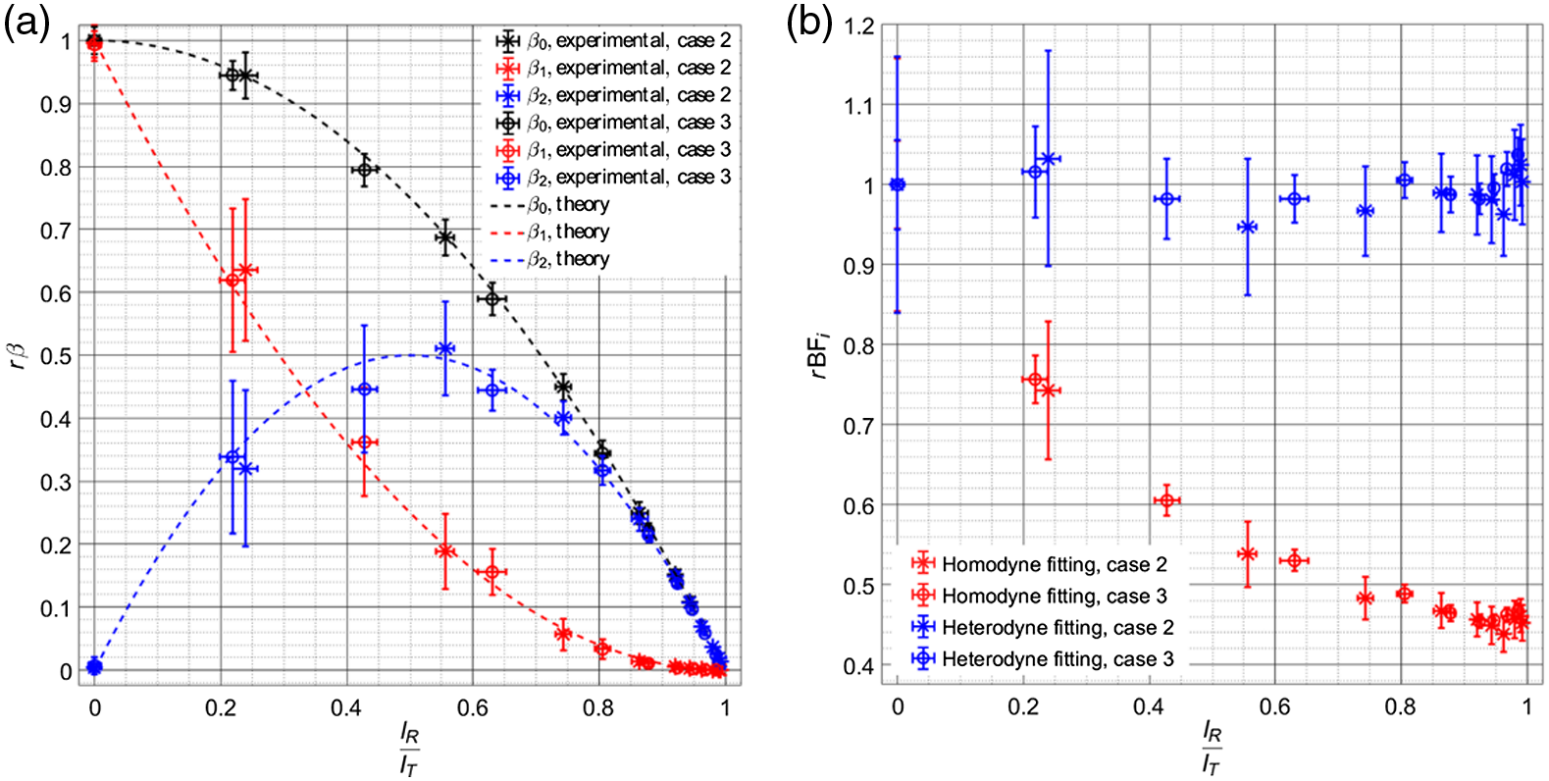
(a) Comparison of the normalized beta values over a range of fractional reference intensities. $\beta_{0}$ (black) represent the beta value fitted from the homodyne form, $\beta_{1}$ (red) represents the weighting of the quadrative component of $g_{1}(\tau )$, and $\beta_{2}$ (blue) represents the weighting of the linear term of $g_{1}(\tau )$. Estimates across different measurements (cases 2 and 3 in Table 1) fall close to the expected value of the given parameters. (b) Relative ${\mathrm{BF}}_{i}$ estimated from measurements across a range of fractional reference intensities. When fitting is done with the heterodyne DCS form (blue), the relative ${\mathrm{BF}}_{i}$ can be seen to cluster around 1, indicating a correct fit, while the homodyne DCS fit (red) of heterodyne data gives a ${\mathrm{BF}}_{i}$ ∼0.5 times the initial measurement, showing the slowing of the decay of the heterodyne autocorrelation function.

Quantification of the coherence weighting parameters, $\beta_{1}$ and $\beta_{2}$, was done using the data from cases 2 and 3 in Table 1. The values of $\beta_{1}$ and $\beta_{2}$ fitted from the autocorrelation functions measured at different fractional reference intensities normalized by the homodyne β value can be seen in Fig. 2(a). The relative ${\mathrm{BF}}_{i}$ values fitted from the autocorrelation functions are shown in Fig. 2(b). In each of these subfigures, the fitting given by the homodyne autocorrelation form is also shown to compare the suitability of the proposed model for fitting these data. Relative ${\mathrm{BF}}_{i}$ values have been normalized by the absolute ${\mathrm{BF}}_{i}$ measurement made by the ISS MetaOx instrument using the standard homodyne DCS approach. Of note, the left-most, $I_{R}/I_{T}=0$ point further verifies the homodyne measurement done with our set up matches the ${\mathrm{BF}}_{i}$ value from the MetaOx measurement. Vertical error bars represent the standard deviation of the fits at 1 Hz over the 180-s measured interval, and the horizontal error bars represent the standard deviation in the fractional reference intensity estimated over the interval using the form, $\overset{¯}{I_{R}/I_{T}}=(\overset{¯}{I_{T}}-\langle I_{S}\rangle )/\overset{¯}{I_{T}}$, where $\overset{¯}{I_{R}/I_{T}}$ is the fractional reference power during the time interval estimated by subtracting the homodyne measurement average count rate, $\left\langle I_{S}\right\rangle$, from the average count rate in the interval, $\overset{¯}{I_{T}}$, then dividing by the average measured count rate. These bars are included to explain a possible source of the variance in the fitted parameters, as variations in the fractional reference intensity contribute to changes in the weighting of the two terms.

From the plots, it can be seen that the estimated weighting values fall closely to the predicted values, and the ${\mathrm{BF}}_{i}$ estimated using the heterodyne fitting form gives the same results across the range of explored reference intensities, showing the suitability of this model to fitting the heterodyne data in this intermediate regime.

### 3.2 Signal-to-Noise Ratio of $g_{1}(\tau )$ and the Coefficient of Variation in the Time Course of ${\mathrm{BF}}_{i}$ for Different Measurement Conditions

**Fig. 3:**
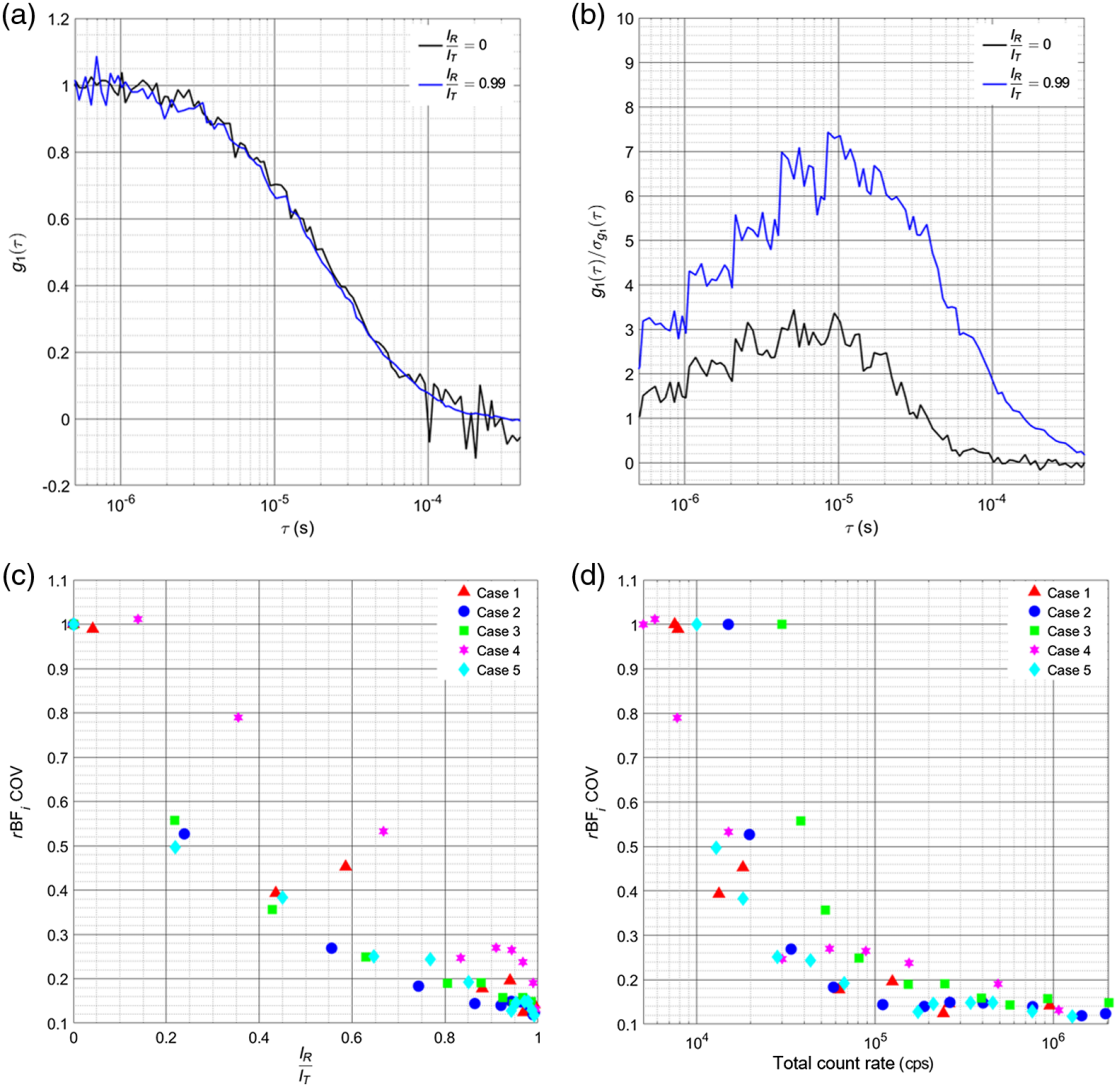
(a) Example of a comparison of $g_{1}(\tau )$ for case 3 between the homodyne measurement ($I_{R}/I_{T}=0$) and the reference-arm-dominated heterodyne measurement ($I_{R}/I_{T}=0.99$), qualitatively showing slightly less variability in the region from 95% of the plateau value to 5% of the plateau value. (b) For the time courses of correlation functions calculated in case 3, the SNR of the $g_{1}(\tau )$ was calculated for the curves seen in (a) are shown. The heterodyne measurement can be seen to improve the SNR of the curve by approximately a factor of 2 at each lag time. To determine how this improvement in SNR affects the fitted ${\mathrm{BF}}_{i}$, plots of the relative coefficient of variation of ${\mathrm{BF}}_{i}$ are shown in (c), showing that for each of the investigated cases, the use of increased reference power reduced variability by up to 80% in the measured ${\mathrm{BF}}_{i}$. (d) The same data shown in (c) are plotted as a function of the summed count rates of the two detectors used for the measurements. The max count rate for an individual detector across all measurements was ∼1  Mcps, allowing for the SPADs to maintain count rate linearity for all measurement cases.

With the model validated, we can now compare the effects of heterodyne detection for different count rates and autocorrelation decay regimes. In Fig. 3(a), an example comparison of $g_{1}(\tau )$ of the homodyne measurement and the reference-arm-dominated heterodyne measurement averaged from the 180-s measured intervals from case 3 is plotted, showing agreement in the shapes of the curves and giving a qualitative idea of the noise of the curves relative to each other. Conversion of $g_{2}(\tau )$ to $g_{1}(\tau )$ is used in this case to ensure that the timing between the homodyne and heterodyne measurements is aligned for effective comparison. In Fig. 3(b), the SNR of $g_{1}(\tau )$, defined as $\frac{g_{1}(\tau )}{\sigma_{g_{1}}(\tau )}$, where $\sigma_{g_{1}}(\tau )$ is the standard deviation of the individual $g_{1}(\tau )$ curves at each lag time calculated at 1 Hz for the 180-s measurement period, is plotted for the homodyne measurement and the reference-arm-dominated heterodyne measurement, showing the heterodyne measurement improves the SNR of the curve.

To show how this increase in SNR affects the variability of the ${\mathrm{BF}}_{i}$ fit, the relative coefficient of variation calculated from correlation functions generated at 1 Hz is plotted for each experimental condition in Fig. 3(c). The relative value for each case is calculated as $\frac{\mathrm{COV}(\frac{I_{R}}{I_{T}})}{\mathrm{COV}(0)}$. These results are also given as a function of the total summed counts between the two detectors, shown in Fig. 3(d).

The increase in SNR of the autocorrelation curve observed in Fig. 3(b) can be seen to translate directly to the decrease in variability observed in the ${\mathrm{BF}}_{i}$ fitting shown in Figs. 3(c) and 3(d). A nearly monotonic decrease in the coefficient of variation relative to the homodyne measurement can be seen for all cases as the fractional reference intensity was increased. This 80% benefit seems to come from both an increase in the SNR of the curve, as the number of relevant signal photons relative to the noise of the autocorrelation, and the slowing of the decay of the autocorrelation function and moving it into a less noisy region of the curve.

### 3.3 Detector Nonidealities, Effects on the Autocorrelation, and Benefits Observed Utilizing Heterodyne Detection

**Fig. 4:**
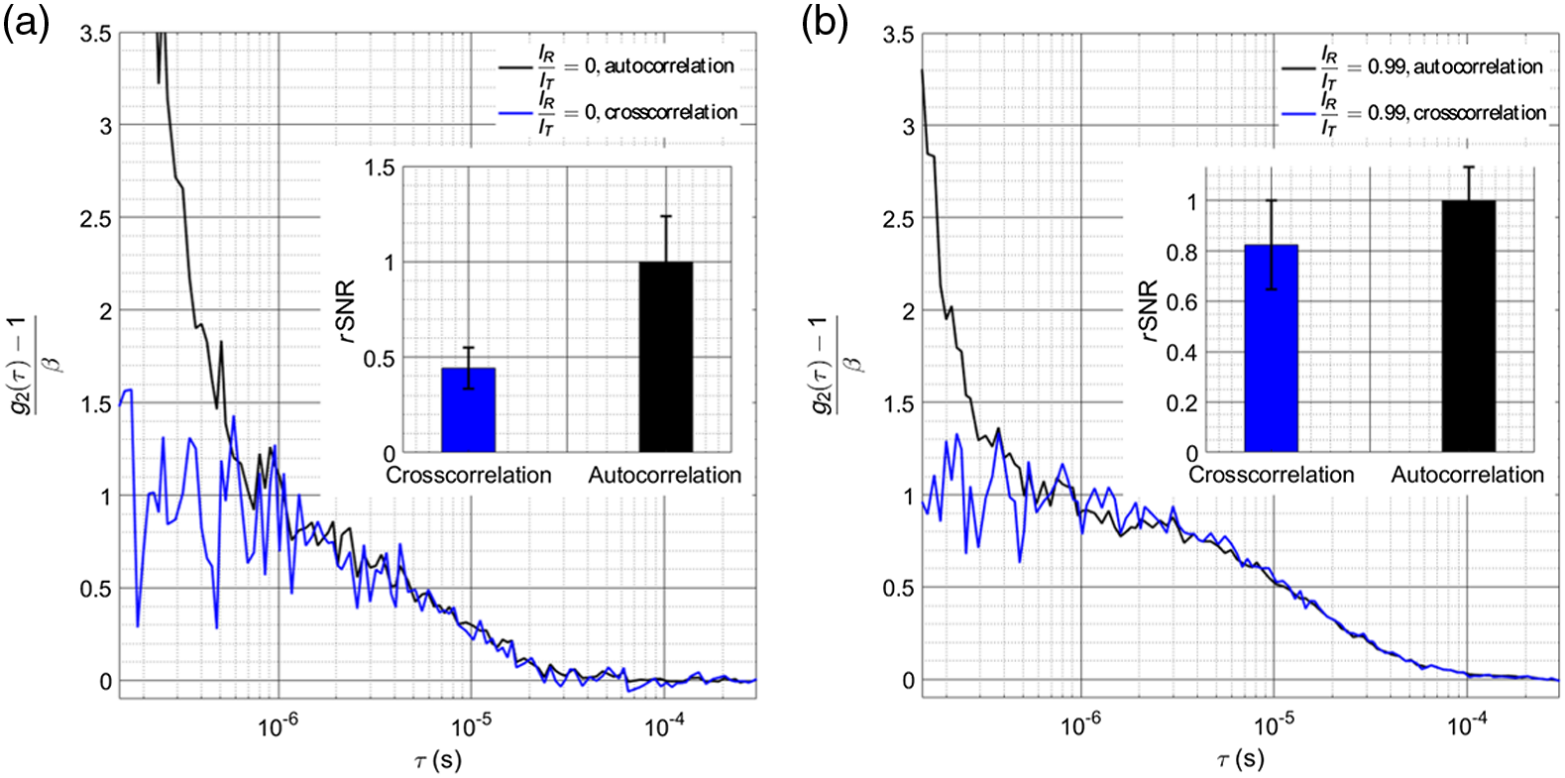
(a) Comparison of the homodyne autocorrelation and cross correlation functions for case 5, including early lag times not typically used for fitting for the DCS ${\mathrm{BF}}_{i}$, to examine the height of the peak caused by the afterpulsing. The typical fitting region of the autocorrelation curve typically begins ∼1  μs, which would reject a large portion of the afterpulsing tail. The inset bar chart shows the relative SNR of the cross correlation and autocorrelation curves, normalized by the SNR of the autocorrelation curve averaged from 1 to 10  μs from correlation functions calculated at 0.1 Hz. (b) When the reference power is increased, the reduction in variability of the plateau of $g_{1}(\tau )$ can be seen in the cross correlation method, and the peak of the afterpulsing is reduced in the autocorrelation method. Further, the slower decay of the reference-arm-dominated measurement pushes the decay to longer lag times, moving it further away from the tail of the afterpulsing. The inset bar chart shows the relative SNR of the cross correlation and autocorrelation curves, normalized by the SNR of the autocorrelation curve.

SPAD detectors are not ideal detectors and have noise characteristics, including dark counts and afterpulsing, that can be managed through the use of heterodyne detection. Dark counts are randomly spaced in time and do not contain a significant temporal coherent behavior. Their main effect on the autocorrelation is to reduce the coherence parameter, β, as only part of the total number of counts measured are coming from the sample. Afterpulsing counts, on the other hand, do have a significant, temporally coherent behavior. To address the challenge in this work, a cross correlation approach has been adopted, effectively turning the correlated afterpulsing counts into uncorrelated dark counts. Though this presents a higher cost for each detection channel (2 versus 1 SPADs), multiple channels are routinely employed to improve SNR at long source–detector separations.^15^^,^^28^ For example in case 5, combining a low count rate experiment and a faster decay of $g_{2}(\tau )$, a comparison of the correlation curves from the 180-s measurement interval generated through the cross correlation and autocorrelation calculations can be seen in Figs. 4(a) and 4(b). Autocorrelations are calculated from the sum of photon counts collected from both detectors. Although the SNR of $g_{2}(\tau )$ calculated by the autocorrelation is higher in the fitting region typically used in DCS, as shown in Fig. 4, the large tail of afterpulsing that is still present has the potential to distort the fitting.

From these results, it can be seen that for low count rate measurements at long source–detector separations, afterpulsing of the SPAD detectors could provide a large, systematic error in the fitted ${\mathrm{BF}}_{i}$ value if time lags influenced by the tail of the afterpulsing are used. This issue could be compounded by the use of the earlier lag times to improve sensitivity to brain blood flow.^15^ The cross correlation used in this work allowed for the characterization of the benefits of the heterodyne approach while avoiding the influence of afterpulsing. Though the SNR of the cross-correlation measurement is reduced relative to the autocorrelation measurement, for fast flows where using early lag times is necessary to properly estimate the flow, the reduction in the tail of the afterpulsing is extremely helpful in accurate quantification and especially relevant in low count rate measurements, as seen in Fig. 4(a). The use of heterodyne detection is shown to be beneficial in dealing with the influence of detector nonidealities by increasing the absolute intensity of the speckle fluctuations to drown out dark counts and afterpulsing counts, and by moving the decay of the autocorrelation function to longer taus, moving the decay away from the tail of the afterpulsing. In addition to drowning out detector intrinsic noise counts, another major benefit of utilizing heterodyne detection is the ability to make robust measurements in the presence of excessive environmental noise counts. As demonstrated by Zhou et al.,^18^ the use of heterodyne detection made the measurement of the ${\mathrm{BF}}_{i}$ insensitive to the presence of room light. This capability is important to improve the robustness of the blood flow measurements in nonideal measurement conditions outside of a laboratory environment, such as in the operating room during surgery or at the bedside in the clinic. An extension of this benefit is the use of less ideal detectors for DCS. Detectors with low dark count rates (<100  cps) were used in this work for characterization, but higher dark count rates can be tolerated due to the amplification of the speckle signal. Equation (3) is presented here under the assumption, for simplicity, that all collected counts are related to either the sample or reference arms, though it can be adapted to include the dark counts of the detector and room light, given as

$$g_{2}(\tau )=1+\frac{1}{M}\{\frac{{\left\langle I_{S}(t)\right\rangle }^{2}}{{\left[\langle I_{S}(t)\rangle +\langle I_{R}(t)\rangle +\langle I_{N}(t)\rangle \right]}^{2}}g_{1}^{2}(\tau )+\frac{2\langle I_{S}(t)\rangle \langle I_{R}(t)\rangle }{{\left[\langle I_{S}(t)\rangle +\langle I_{R}(t)\rangle +\langle I_{N}(t)\rangle \right]}^{2}}g_{1}(\tau )\},$$ (6)

where M is the number of detected modes and $I_{N}$ is the intensity of the noise. For this formulation of the autocorrelation function, the sum of the coherence parameters for the linear and quadratic terms can be seen to increase to a maximum when $I_{R}=I_{N}$, as has been previously demonstrated.^24^ When $I_{R}\gg I_{N}$ and $I_{R}\gg I_{S}$, the expression effectively reduces to the case with no noise, where $g_{2}(\tau )=1+\frac{2\left\langle I_{S}(t)\right\rangle }{M\langle I_{R}(t)\rangle }g_{1}(\tau )$. This characteristic can allow for the use of less expensive detectors with less ideal noise characteristics, which could offset the cost of using two detectors to compute the cross correlation.

### 3.4 Practical Considerations for Implementation

Benefits from the use of the technique can be seen even in cases where count rates would be sufficient in the homodyne measurement to accurately resolve blood flow (case 3), and so use of heterodyne detection as an add-on to commonly used DCS systems provides an advantage to the achievable SNR. While in the results presented here the changes in detected intensity at each source–detector distance were caused by changes in laser power sent to the sample, this does not necessarily reflect the reality of *in vivo* measurements, where higher tissue absorbance will attenuate the signal. The attenuation is more severe for longer path lengths, which results in a slowing of the autocorrelation decay. In all cases examined here, we effectively present the worst-case scenario for the reduced count rate measurements, where the decay of the autocorrelation does not slow down but the count rate drops appreciably, and show that heterodyne detection still gives significant improvement to the precision of the ${\mathrm{BF}}_{i}$ measurements. Conversion to heterodyne can be achieved without the need to modify an existing homodyne DCS system. The implementation of the technique requires just splitting part of the source laser and recombining it with detected light, which can be achieved through fiber couplers placed within or external to the containment boxes of standard systems. The use of fiber couplers does not necessarily represent a great increase in cost relative to the full system, ∼$400  per channel, and does not require a great deal of physical space within the enclosures to implement. Though the calculation of $g_{2}(\tau )$ was done using the cross correlation between two detectors, representing a greater cost, taking the autocorrelation of the single detector could prove viable for some cases, as shown in Fig. 4. An aside in the implementation used here to characterize the iDCS system, single-mode fiber was used in as much of the system as possible to reduce the effects of motion on the transfer matrix of the fiber couplers. Motion of multimode fibers causes a shift in the mode transfer matrix, which can destabilize the reference and source intensities, and add distortions to the autocorrelation function that reflect the motion of the fibers. This effect can be minimized by the use of single-mode fiber and single-mode fiber couplers. The laser used here was coupled initially to multimode fiber, allowing for improved power coupling at a given price point for the system as a homodyne DCS instrument, and to allow for efficient coupling, the 99/1% fiber coupler was also multimode fiber. To reduce possible disruption, the fiber was tightly secured, and because the measurements were done on phantoms, this proved sufficient to stabilize the coupling. For *in vivo* measurements though, subject motion may require the use of single-mode fiber coupled lasers to allow for single-mode splitters. On the source side of the system, given that most of the detected light comes from the reference arm, stability of the laser source is essential to avoid distorting the measured correlations (more so than for homodyne measurements). DCS lasers are typically quite stable, and so this requirement should not greatly impact implementation with current systems, as was done in this study. Nonlinearity of SPAD detectors as a function of the count rate could limit the amplification achievable while still leaving the autocorrelation undistorted. In this work, the maximum count rate used was ∼1  Mcps per channel, as seen in Fig. 4(b), which allowed for a count rate linearity, defined as $100*(1-t_{d}*CR)$, where $t_{d}$ is the detector dead time and CR is the count rate, of 97.8% ($t_{d}=22\;\mathrm{ns}$). The maximum count rate for each case explored gave a range of maximum $I_{R}/I_{T}$ between 0.985 (case 3) and 0.998 (case 4), allowing for close to shot-noise limited performance. Improvement of SNR is presented across all measurement conditions, though care should be taken to maintain the linear response of the detector and reduce dead time effects to not distort the calculated autocorrelation. Fitting the heterodyne correlation function could also present an increase in the variability of the estimated ${\mathrm{BF}}_{i}$, as there are now three parameters to fit ($\beta_{1}$, $\beta_{2}$, and ${\mathrm{BF}}_{i}$). The uncertainty is magnified when the linear and quadratic terms of $g_{1}(\tau )$ are approximately equal, seen in Figs. 2(a) and 2(b) for $I_{R}/I_{T}$ between 0.2 and 0.5. This uncertainty can be managed by increasing the intensity of the reference arm, allowing the linear term of $g_{1}(\tau )$ to dominate. In general, an optimization of reference arm power and source–detector separation could be fairly easily implemented to improve sensitivity to cerebral blood flow, increase the SNR of the measurement, and maintain detector linearity. Of note, the use of photon counting detectors instead of the camera-based approach allows the use of existing hardware correlators instead of requiring software postprocessing and also results in more manageable data volumes, especially for longer recordings.

## 4 Conclusion

In this work, characterization of heterodyne detection of DCS signals, a technique we have called interferometric diffuse correlation spectroscopy (iDCS), was performed for a range of conditions. Agreement with theoretical predictions was shown in measurements with good SNR, and accurate ${\mathrm{BF}}_{i}$ values were extracted across a range of fractional reference intensity values. iDCS has the potential to improve the accuracy of the estimation of blood flow by reducing the variability of the fitted ${\mathrm{BF}}_{i}$ value by improving the SNR of the autocorrelation curve by addressing low count rate, moving the fitting to a less noisy region of time lags, and compensating for detector nonidealities. These features are especially relevant for DCS measurements of cerebral blood flow, which require long (≥2.5  cm) source–detector separations to have sufficient sensitivity to the brain, and could benefit from this approach. The practical considerations of implementing this technique with existing DCS devices were discussed, and based on the relative ease by which systems could be upgraded to include iDCS channels, could enable more accurate and sensitive, noninvasive cerebral blood flow measurements at the bedside.
